# Solitary Fibrous Tumor of the Kidney Developing Local Recurrence

**DOI:** 10.1155/2016/2426874

**Published:** 2016-04-28

**Authors:** Wataru Usuba, Hideo Sasaki, Hidekazu Yoshie, Kazuki Kitajima, Hiroya Kudo, Ryuto Nakazawa, Yuichi Sato, Masayuki Takagi, Tatsuya Chikaraishi

**Affiliations:** ^1^Department of Urology, St. Marianna University, 2-16-1 Sugao, Miyamae, Kawasaki 216-8511, Japan; ^2^Department of Pathology, St. Marianna University, 2-16-1 Sugao, Miyamae, Kawasaki 216-8511, Japan

## Abstract

Solitary fibrous tumor (SFT) of the kidney is a rare entity and usually displays a favorable prognosis. We herein report a second case of renal SFT developing local recurrence. A 50-year-old man was referred to our hospital because of a left renal mass. An abdominal CT detected a large renal tumor and radical nephrectomy was performed with a possible diagnosis of renal cell carcinoma. The resected tumor size was measured at 17 × 11 × 8 cm. Grossly, necrosis was observed in central lesion of the tumor but hemorrhage was not observed. Microscopically, the tumor consisted of spindle-shaped cells with scant cytoplasm accompanied by hyalinized collagenous tissue, which displayed hemangiopericytomatous patterns. The cellularity was normal and nuclear pleomorphism was not observed. Ki-67 labeling index was less than 3%. The pathological diagnosis of SFT was made without obvious malignant findings. Three years after the surgery, a follow-up CT scan detected a mass lesion in the tumor bed. Surgical resection was performed and the resected tumor was compatible with local recurrence of the SFT without obvious malignant findings. Renal SFT should be carefully monitored even in the absence of obvious malignant findings.

## 1. Introduction

Solitary fibrous tumor (SFT) is a clinical entity that was first reported as a tumor of the pleura in 1931 and usually arises in the pleura [1]. SFT is a rare spindle cell neoplasm and it is postulated that the tumor originated from mesenchymal tissue [2]. Histologically SFT shows hemangiopericytoma-like growth pattern and immunohistochemical staining for CD-34 and Bcl-2 is helpful for diagnosing the SFT. SFT typically is strong and diffusely positive for CD-34 and 70% of the SFT is positive for Bcl-2 [3]. The disease commonly arises from the thoracic cavity, yet it may arise from other sites including the kidney [2]. SFT of the kidney is an extremely rare and generally indolent tumor, unlikely to recur locally or distantly. Up to the present, only 81 cases of occurring renal SFT have been reported. SFT of the kidney usually displays a favorable prognosis and only two cases were reported to develop a distant metastasis. Furthermore local recurrence of SFT of the kidney had been reported in only one case [4]. Herein, we describe the second case of local recurrence of renal SFT after radical.

## 2. Case Presentation

**Figure 1:**
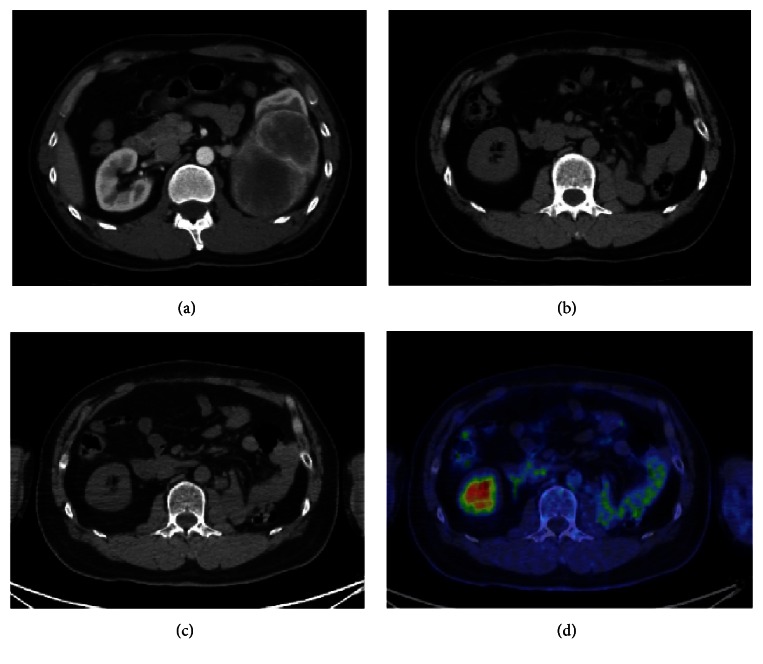
Radiological findings of the renal SFT. Enhanced abdominal CT revealed 17 × 11 × 8 cm tumor located in the left kidney (a). Follow-up plain CT revealed suspicions of recurrent tumor (1 × 0.7 cm) in the tumor bed at 3 years after the nephrectomy (b). Three months after the CT, which detected suspicions of recurrent tumor, follow-up CT scan and PET-CT were performed. (c, d) The mass lesion was increased in size (1.7 × 1.1 cm).

**Figure 2:**
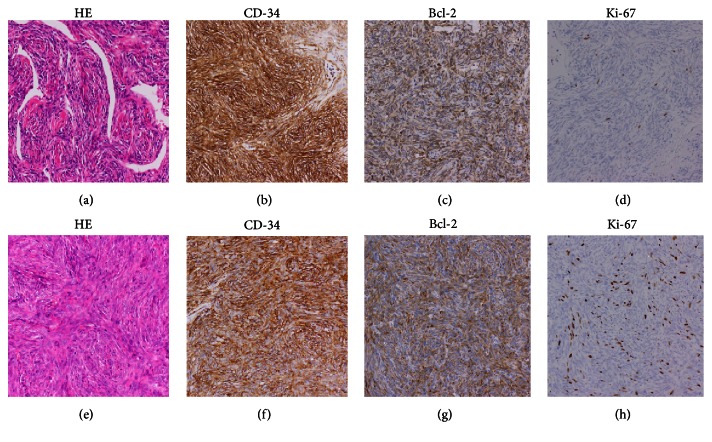
Histological findings of the solitary fibrous tumor. The primary tumor displayed hemangiopericytomatous patterns ((a), HE ×40). Immunohistochemical staining of the primary tumor was positive for CD-34 and Bcl-2 ((b) and (c), ×40) and Ki-67 labeling index was less than 3% ((d), ×40). Cellularity was increased in the tumor that recurred at the hilar portion of the kidney (e). Immunohistochemical staining for CD-34 and Bcl-2 was positive ((f) and (g), ×40). Ki-67 labeling index was less than 15% ((h), ×40).

**Figure 3:**
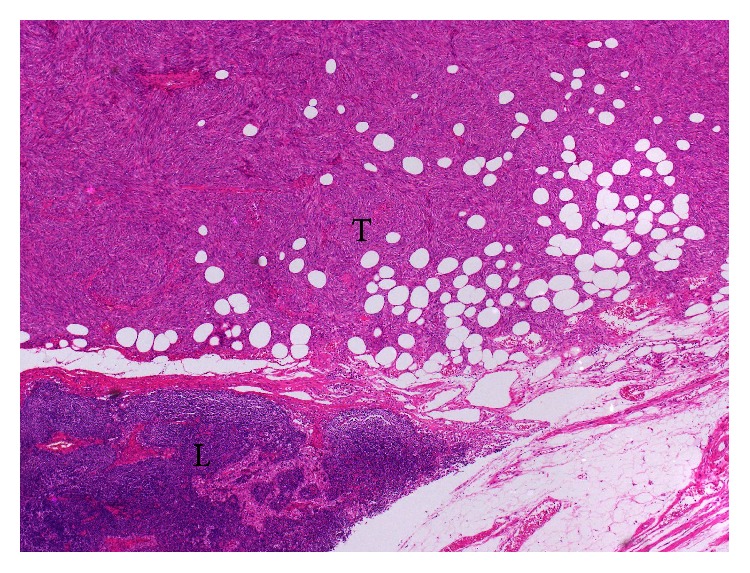
Histological finding of local recurrence of the solitary fibrous tumor. The recurrent tumor was developed from an extra nodal connective tissue (×10). L: lymph node; T: tumor.

A 50-year-old male was referred to our hospital because of a left renal mass, which had been incidentally detected by ultrasonography performed in a routine health check-up. A physical examination and blood chemical analysis were normal. Subsequent computed tomography (CT) scan detected a well-enhanced large left renal tumor (Figure 1(a)). He was diagnosed with left renal cell carcinoma preoperatively, and radical nephrectomy was performed. Grossly, the tumor was measured at 17 × 11 × 8 cm, was well-circumscribed, and displayed necrosis with a gray-white cut surface. Hemorrhage was not observed. Microscopically, the tumor was composed of spindle-shaped cells, which displayed hemangiopericytomatous patterns (Figure 2(a)). The tumor displayed normal cellularity without nuclear pleomorphism. Mitotic count was less than 1 per 10 high power fields. Immunohistochemical staining was positive for CD-34 (Figure 2(b)), Bcl-2 (Figure 2(c)), CD-99, and STAT-6, all of them representing conventional immunohistochemical markers for SFT. Meanwhile, SMA stain was negative and Ki-67 labeling index was less than 3% (Figure 2(d)). Thus, he was histologically diagnosed with SFT of the kidney without obvious malignant findings. Postoperatively, follow-up CT examination was performed regularly every 3-4 months. Three years after the operation, a mass lesion was detected in the tumor bed (Figure 1(b)). The mass lesion was increased in size after 3 months (Figure 1(c)). Fluorodeoxyglucose (FDG) positron emission tomography (PET) was ordered but the tumor did not accumulate FDG (Figure 1(d)). Nonetheless, as a local recurrence or lymph node metastasis could not be denied, we planned a surgical removal of the tumor. Although the recurrent tumor displayed spindle-shaped cells with hemangiopericytomatous patterns as in the original tumor, the cellularity was increased and cytological atypia was observed (Figure 2(e)). These results suggested an increased malignant potential of the tumor, but mitotic count was less than 4 mitoses per 10 high power fields. Immunohistochemical staining for CD-34 (Figure 2(f)), Bcl-2 (Figure 2(g)), and CD-99 all remained positive. Ki-67 labeling index was less than 15% (Figure 2(h)) and SMA stain was positive in the resected tissue from the tumor bed. Although an increased malignant potential was suggested, pathological findings did not meet the diagnostic criteria of malignant SFT [5]. The recurrent tumor was developed from an extra nodal connective tissue not from the lymph node (Figure 3). Therefore, we diagnosed local recurrence of renal SFT without evidence of obvious malignant findings. Twelve months after the second operation, the patient is followed up on the outpatient basis with no evidence of local recurrence or distant metastasis.

## 3. Discussion

In 1931, SFT was firstly reported as a tumor of the pleura [1]. It is a rare tumor comprising spindle-shaped cells, which might originate from mesenchymal tissue [2]. Although SFT is commonly thought of as an intrathoracic tumor, it could arise from extrathoracic organs, including the kidney [2]. Surgical resection is a standard treatment and complete resection can be associated with a favorable prognosis, even if the SFT is histologically diagnosed as malignant [4, 6].

SFT of the kidneys is a rare neoplasm, and Sasaki et al. reviewed the 68 cases of SFT in 2013 [7], and additional 13 cases were reported up to now. All reported cases, including our case, are summarized in Table 1. Most of the tumors were incidentally found with no apparent clinical symptoms. Preoperatively, most of them were diagnosed as renal cell carcinoma, and 72 out of 82 cases underwent radical nephrectomy. Mean age at diagnosis was 52.8 ± 17.7 (3–85) years and mean tumor size was 9.5 ± 6.2 (2–29) cm. Histologically, 68 tumors showed a benign appearance, whereas 11 cases exhibited a malignant one. Most patients displayed a favorable prognosis with no evidence of recurrence during the follow-up period, ranging from 0.1 to 96 months. Only 4 patients experienced recurrence; 2 patients developed distant metastasis; and 2 patients, including the present case, developed local recurrence.

As SFT commonly expresses CD-34, Bcl-2, and CD-99 [8], these surface antigens can serve as useful diagnostic markers [8]. And negativity in CD-34 and Bcl-2 reportedly represents increased malignant potential [8, 9]. Fine et al. documented a case of malignant renal SFT without expressing CD-34, which developed distant metastasis four months after surgery [10]. We also reported a similar case previously, which did not express CD-34 and went on to metastasize to the lung and liver [7]. In that case, half of the cross section area of the primary tumor was positive for CD-34, while the remaining area was negative for it. The patient developed distant metastases 8 years after nephrectomy. Resection of the metastatic tumors had revealed that CD-34 was totally absent in the tumors. Thus, the loss of CD-34 staining in SFT of the kidney may promote tumor metastasis to other organs [7]. Similarly to CD-34 staining, Bcl-2 staining was commonly observed in SFT and the loss of Bcl-2 staining was reported to be associated with malignant potential in retroperitoneal SFTs [9].

On the contrary, malignant potential is rather low in the present case, which developed local recurrence 3 years after nephrectomy. In this case, no obvious malignant findings were observed in either primary or recurrent tissue from the tumor bed. Furthermore, CD-34 and Bcl-2 were positive in the primary tumors and remained positive in the recurrent tissue. It seems that the local recurrence does not necessarily accompany the loss of expression of CD-34 and Bcl-2, and another explanation for unpredicted local recurrence would be incomplete resection at surgery [5]. However, from a different standpoint, the tumor in the present case may have had a great tendency to local recurrence, as the tumor accompanies multiple clinical features such as extrathoracic location, large tumor size, increased cellularity, and presence of necrosis among the risk factors for local recurrence described by Jason et al. [11].

Overall, we believe that there is no strict dichotomy between benign and malignant SFTs and that all tumors likely have some degree of metastatic potential, albeit quite low. Therefore, although renal SFT is thought to be a benign tumor, an adequate follow-up period is required to evaluate the precise clinical outcome of renal SFT, and the follow-up period in this report of 82 patients may not be sufficient (Table 1). Furthermore, most reported renal SFTs were large in size at the diagnosis and it might be leading cause of missing the malignant features in whole tumor tissue. We should also concern this issue for evaluating the real feature of renal SFTs in future.

FDG accumulation was not observed within the tumor on FDG-PET. To date, there is no reported association between SFTs and FDG accumulation, and our result suggests that PET-CT may be invalid. Further detailed examination is also required to clarify this point.

In conclusion, a case of SFT of the kidney exhibiting local recurrence was reported. In our case, no obvious malignant findings were observed in either the primary tumor or the recurrent tumor. Loss of expression in CD-34 and Bcl-2, which is closely associated with malignant potential, was not observed. Although SFT of the kidney usually displays a favorable clinical course, careful and sufficient follow-up may be required even in the absence of malignant findings.

## Figures and Tables

**Table 1:** Clinicopathological findings of renal solitary fibrous tumors in the literature.

Case	Year	Age	Sex	Symptom	Side	Affected site	Tumor size (cm)	Treatment	Histology	Follow-up (month)	Outcome	CD-34^*∗*^	Authors and journals
1	1996	48	M	Back pain and macrohematuria	R	Renal capsule	3	Nephrectomy	BEN	0.1	DNOD	POS	Gelb et al. Am J Surg Pathol 20:1288
2	1996	45	F	Incidental	R	Kidney	6	Nephrectomy	BEN	8	NED	POS (2/3)	Fain et al. J Urol Pathol 4:227
3	1996	46	F	Incidental	R	Kidney	7.2	Nephrectomy	BEN	33	NED	POS (2/3)	Fain et al. J Urol Pathol 4:227
4	1996	51	M	Incidental	L	Kidney	4.5	Nephrectomy	BEN	2	NED	POS (2/3)	Fain et al. J Urol Pathol 4:227
5	1997	33	F	Abdominal pain	R	Peripelvis	3.5	Nephrectomy	BEN	89	NED	POS	Fukunaga et al. Histopathology 30:451
6	1997	36	F	Abdominal pain	L	Peripelvis	2	Nephrectomy	BEN	12	NED	POS	Fukunaga et al. Histopathology 30:451
7	1998	59	M	Incidental	L	Renal capsule	NA	Nephrectomy	BEN	NA	NA	POS	Ookouci S et al. Jpn J Radiol 58:539
8	1998	57	M	Incidental	L	Kidney	7	Tumorectomy	BEN	NA	NA	POS	Tanahashi C et al. Proc Jpn Soc Pathol 87:510
9	1999	64	M	Macrohematuria	R	Kidney	4.5	Nephrectomy	BEN	8	NED	POS	Hasegawa et al. Hum Pathol 30:1464
10	1999	71	F	Incidental	L	Kidney	9	Nephrectomy	BEN	NA	NA	NA	Kojima K et al. Jap-Deu Med Beriche 44:185
11	2000	66	F	Abdominal pain and macrohematuria	R	Kidney	9	Nephrectomy	BEN	9	NED	POS	Leroy et al. Urol Int 65:49
12	2000	72	F	NA	L	Kidney	8	Nephrectomy	BEN	10	NED	POS	Morimitsu et al. APMIS 108:617
13	2000	56	F	Incidental	L	Renal capsule	5	Tumor resection	BEN	NA	NA	NA	Ikeda A et al. J Hiroshima Med Assoc 53:640
14	2001	70	M	Incidental	R	Renal pelvis	6	Nephrectomy	BEN	60	NED	POS	Yazaki et al. Int J Urol 8:504
15	2001	28	F	Abdominal pain	L	Kidney	15	Nephrectomy	BEN	12	NED	POS	Cortes-Gutierrez et al. J Urol 166:60
16	2001	41	M	Macrohematuria	L	Kidney	14	Nephrectomy	BEN	48	NED	POS	Wang J et al. Am J Surg Pathol 25:1194
17	2001	72	M	Abdominal discomfort	R	Kidney	13	Nephrectomy	BEN	5	NED	POS	Wang J et al. Am J Surg Pathol 25:1194
18	2002	57	M	Incidental	L	Kidney	6	Nephrectomy	BEN	NA	NA	POS	Miyazaki N et al. Jpn Red Cross Med J 54:182
19	2002	58	M	Incidental	L	Kidney	NA	Nephrectomy	BEN	9	NED	NA	Inokawa E J Hiroshima Med Assoc 55:1057
20	2002	31	F	Flank pain	R	Kidney	8.6	Nephrectomy	BEN	8	NED	POS	Magro G Pathol Res Pract 198:37
21	2003	64	F	Microhematuria	R	Kidney	4	Nephrectomy	BEN	7	NED	POS	Li S et al. Hinyokika Kiyo 49:121
22	2003	51	F	NA	R/L	Kidney	25 and 2	Tumor resection	BEN	NA	NA	NA	Llarena Ibarguren et al. Arch Esp Urol 56:835
23	2003	35	M	NA	R	Kidney	17	Nephrectomy	BEN	6	NED	NA	Durand X et al. Prog Urol 13:491
24	2003	60	F	NA	R	Kidney	11	Nephrectomy	BEN	48	NED	NA	Bugel H et al. Prog Urol 13:1397
25	2004	67	M	Incidental	L	Kidney	4.5	Tumorectomy	BEN	5	NED	POS	Toriyama S et al. Hinyokika Kiyo50:138
26	2004	83	M	NA	R	Kidney	9	Nephrectomy	BEN	18	NED	POS	Gres P et al. Prog Urol 14:65
27	2004	53	M	Flank pain and swelling	R	Renal capsule	14	Tumor resection	BEN	36	DNOD	POS	Kunieda K et al. Surg Today 34:90
28	2004	59	M	Incidental	L	Renal capsule	6.8	Nephrectomy	BEN	48	NED	POS	Yamada H et al. Pathol Int 54:914
29	2005	29	NA	Incidental	NA	Kidney	2.2	Nephrectomy	BEN	NA	NA	POS	Pierson DM et al. Mod Pathol 18:159
30	2005	NA	NA	Incidental	NA	Kidney	NA	Nephrectomy	BEN	NA	NA	POS	Pierson DM et al. Mod Pathol 18:160
31	2005	NA	NA	Incidental	NA	Kidney	NA	Nephrectomy	BEN	NA	NA	POS	Pierson DM et al. Mod Pathol 18:161
32	2005	NA	NA	Incidental	NA	Kidney	NA	Nephrectomy	BEN	NA	NA	POS	Pierson DM et al. Mod Pathol 18:162
33	2005	NA	NA	Incidental	NA	Kidney	NA	Nephrectomy	BEN	NA	NA	POS	Pierson DM et al. Mod Pathol 18:163
34	2005	NA	NA	Flank pain	NA	Kidney	NA	Nephrectomy	BEN	NA	NA	POS	Pierson DM et al. Mod Pathol 18:164
35	2005	79	NA	Flank pain	NA	Perirenal	10.1	Nephrectomy	BEN	NA	NA	POS	Pierson DM et al. Mod Pathol 18:165
36	2005	51	F	Flank pain	NA	Renal capsule	10	Nephrectomy	BEN	NA	NA	POS	Yamaguchi T Urology 65:175
37	2005	51	F	Fever elevation	R	Renal capsule	13	Nephrectomy	BEN	NA	NA	POS (focal)	Jhonson TR et al. J Comput Assist Tomogr 29:481
38	2005	83	F	Incidental	L	Kidney	11	Nephrectomy	BEN	NA	NA	POS	Kawagoe M Nishinihon J Urol 67:568
39	2006	76	M	Incidental	L	Kidney	12	Nephrectomy	MAL	4	Lung metastasis	POS (benign site)	Fine SW et al. Arch Pathol Lab Med 130:857
40	2006	18	F	Flank pain	L	Kidney	3	Nephrectomy	BEN	15	NED	POS	Koroku M et al. Hinyokika Kiyo 52:705
41	2006	4	M	NA	R	Kidney	8	Nephrectomy	BEN	NA	NA	NA	Provance et al. Clin Pediatr 45:871
42	2006	85	M	Flank pain	L	Kidney	4.5	Nephrectomy	BEN	NA	NA	POS	Kohl SK et al. Arch Pathol Lab Med 130:117
43	2006	54	M	Incidental	R	Kidney	NA	Nephrectomy	BEN	16	NED	POS	Tanaka M et al. Hinyokika Kiyo 52:79
44	2006	36	M	Flank pain	R	Kidney	NA	Nephrectomy	BEN	NA	NA	NA	Alvarez Mugica M et al. Arch Esp Urol 59:195
45	2007	26	M	Incidental	R	Kidney	7	Nephrectomy	BEN	6	NED	POS	Constantinidis C et al. The Can J Urol 14:3583
46	2007	70	M	Flank pain and macrohematuria	L	Kidney	15	Nephrectomy	BEN	6	NED	POS	Znati K et al. Revies in Urol 9:36
47	2007	51	F	Flank pain	L	Kidney	4	Nephrectomy	BEN	10	NED	POS	Bozkurt SU et al. APMIS 115:259
48	2007	66	F	Abdominal mass and macrohematuria	R	Kidney	11	Nephrectomy	BEN	NA	NA	NA	Kakoi N et al. Japn J Urol Surg 20 supple 598
49	2007	60s	M	Incidental	R	Kidney	3	Nephrectomy	BEN	3	NED	NA	Yoshida T et al. Hinyokika Kiyo53:745
50	2008	34	F	Flank pain	L	Kidney	9	Nephrectomy	MAL	21	NED	POS	Magro G et al. APMIS 115:1020
51	2008	67	M	Macrohematuria	L	Kidney	7	Nephrectomy	BEN	10	NED	POS	Amano T et al. Hinyokika Kiyo54:357
52	2008	44	F	Incidental	L	Kidney	5.8	Nephrectomy	BEN	40	NED	POS	Hirabayashi J et al. Hinyokika Kiyo54:357
53	2009	75	F	Incidental	L	Kidney	4.5	Nephrectomy	BEN	9	NED	POS	Hirano D et al. Mod Mol Morphol 42:239
54	2009	64	F	Cough	L	Kidney	2.5	Biopsy	BEN	12	NED	POS	Petrella F et al. Minerca Chir 64:669
55	2009	35	M	Incidental	R	Kidney	8	Partial nephrectomy	BEN	NA	NA	POS	Makris A et al. Can J Urol 16:4854
56	2009	72	F	Abdominal mass	L	Kidney	19	Nephrectomy	MAL	NA	NA	NA	Marzi M et al. Urologia 76:112
57	2009	76	F	Incidental	R	Kidney	2.5	Nephrectomy	BEN	48	NED	POS	Yoneyama T et al. Hinyokika Kiyo 55:479
58	2009	50	M	Incidental	L	Kidney	5.5	Nephrectomy	BEN	NA	NED	POS	Matsumoto T et al. Japn J Urol Surg 22:230
59	2009	63	M	Incidental	L	Kidney	5.3	Nephrectomy	MAL	NA	NA	POS	Murayama S et al. Japn J Urol Surg 22:230
60	2009	51	F	Incidental	R	Kidney	12	Nephrectomy	BEN	NA	NA	POS	Ogushi S et al. Japn J Urol Surg 22:230
61	2009	75	M	NA	L	Kidney	3	Nephroureterectomy	BEN	NA	NA	POS	Kobori Y et al. Hinyokika Kiyo 55:305
62	2010	39	M	Dysuria	L	Kidney	25	Nephrectomy	BEN	12	NED	POS	Taza L et al. Actas Urol Esp 34:568
63	2010	39	F	Abdominal fullness	L	Kidney	20	Embolization and nephrectomy	BEN	6	NED	POS	Yamaguchi Y et al. Hinyokika Kiyo 56:435
64	2011	44	M	Macrohematuria	L	Kidney	NA	Embolization and nephrectomy	BEN	NA	NA	NA	Saegusa M et al. Nishinihon J Urol 68:187
65	2011	52	F	Abdominal pain	R	Kidney	18	Nephrectomy and thrombectomy	BEN	6	NED	POS	Naveen HN et al. Urol Ann 3:158
66	2011	72	F	Abdominal mass	L	Kidney	19	Nephrectomy	MAL	15	NED	POS (focal)	Marzi M et al. Minerva Urol Nephrol 63:109
67	2011	50	F	Flank pain	R	Kidney	15	Nephrectomy	MAL	30	NED	POS	Tsan-Yu Hsieh Diag Pathol 6:96
68	2012	68	F	Flank pain	NA	Kidney	NA	Nephrectomy	MAL	NA	NA	POS	M. de Martino Aktuel Urol 2012; 43(01):59–62
69	2012	72	M	Flank pain	L	Kidney	7	Nephrectomy	MAL	45	NED	POS	Sfoungaristos S Prague Med Rep/Vol 113 No. 3, 246–250
70	2012	56	M	Shortness of breath	L	Kidney	10, 10	Nephrectomy	MAL	10	NED	POS	G. Zhao et al. Oncology Letters 4:993–995, 2012
71	2013	49	F	Dyspnea	L	Kidney	NA	Nephrectomy	BEN	23	SD	POS	J. Cuello et al. Case Rep Oncol Med 2013; 2013:564980
72	2013	48	M	Abdominal mass	R	Kidney	29	Nephrectomy	BEN	96	NED	POS (55%)	Sasaki H et al. Case Rep Nephrol Urol 3:1–8
73	2013	57	M	Lumbar pain	L	Kidney	14	Nephrectomy	BEN	26	NED	POS	Abdullah D et al. Case Report in Urol 147496:4
74	2013	3	M	NA	NA	Kidney	NA	Nephrectomy	NA	NA	NA	NA	Wu WW et al. Int J Surg Pathol 23(1):34–47
75	2013	49	F	Fever elevation and flank pain	R	Kidney	5	Nephrectomy	BEN	NA	NED	POS	Nazih K et al. Urol Int 2013; 91:373–383
76	2013	43	M	Acute recurrent pancreatitis	NA	Kidney	NA	NA	NA	NA	NA	NA	Patel YA et al. Pancreatology 13(6):631–3
77	2013	30	F	NA	NA	Renal pelvis	NA	Nephrectomy	BEN	NA	NA	NA	Pathak TB et al. JNMA Apr-Jun; 52(190):388–90
78	2014	66	F	Flank mass	R	Kidney	26	Nephrectomy	MAL	9	NED	POS > NEG	Wang et al. Diagnostic Pathol 9:13
79	2014	19	F	Hematuria	L	Kidney	14.5	Embolization and nephrectomy	MAL	30	NED	POS	Ettore M et al. Onco Targets and Therapy Jul 679–685
80	2014	35	F	Back pain	L	Kidney	3	Nephrectomy	BEN	15	NED	POS	Jie Ma et al. Int J Clin Exp Pathol 7(7):4268–4237
81	2014	55	NA	NA	NA	Kidney	NA	Nephrectomy	NA	NA	NA	NA	Tritschler P et al. JBR-BTR Sep-Oct; 97(5):298–300
82	our case	50	M	Incidental	L	Kidney	17	Nephrectomy	BEN	36	LR	POS	

M, male; F, female; NA, not available; R, right; L, left; BE, benign; MAL, malignant; DNOD, died not of disease; NED, no evidence of disease; SD, stable disease; LR, local recurrence; POS, positive.

^*∗*^CD-34 immunoreactivity (the extent of positive area is shown in parentheses, if information is available).
